# Multiresistant Enterobacteriaceae in yellow‐legged gull chicks in their first weeks of life

**DOI:** 10.1002/ece3.8974

**Published:** 2022-06-11

**Authors:** Marion Vittecoq, Lionel Brazier, Eric Elguero, Ignacio G. Bravo, Nicolas Renaud, Alejandro Manzano‐Marín, Franck Prugnolle, Sylvain Godreuil, Thomas Blanchon, François Roux, Patrick Durand, François Renaud, Frédéric Thomas

**Affiliations:** ^1^ Lab. Mivegec University Montpellier CNRS IRD UMR5290 CREES Montpellier France; ^2^ Tour du Valat Research Institute for the Conservation of Mediterranean Wetlands Arles France; ^3^ SYNLAB Midi Montpellier France; ^4^ 27258 Centre for Microbiology and Environmental Systems Science University of Vienna Vienna Austria

**Keywords:** antimicrobial resistance, enterobacteria, *Larus*, third‐generation cephalosporin, whole‐genome sequencing, wild birds

## Abstract

Wild animal species living in anthropogenic areas are commonly carriers of antimicrobial‐resistant bacteria (AMRB), but their role in the epidemiology of these bacteria is unclear. Several studies on AMRB in wildlife have been cross‐sectional in design and sampled individual animals at only one point in time. To further understand the role of wildlife in maintaining and potentially transmitting these bacteria to humans and livestock, longitudinal studies are needed in which samples are collected from individual animals over multiple time periods. In Europe, free‐ranging yellow‐legged gulls (*Larus michahellis*) commonly live in industrialized areas, forage in landfills, and have been found to carry AMRB in their feces. Using bacterial metagenomics and antimicrobial resistance characterization, we investigated the spatial and temporal patterns of AMRB in a nesting colony of yellow‐legged gulls from an industrialized area in southern France. We collected 54 cloacal swabs from 31 yellow‐legged gull chicks in 20 nests on three dates in 2016. We found that AMRB in chicks increased over time and was not spatially structured within the gull colony. This study highlights the complex occurrence of AMRB in a free‐ranging wildlife species and contributes to our understanding of the public health risks and implications associated with ARMB‐carrying gulls living in anthropogenic areas.

## Graphical abstract

We investigated the spatial and temporal patterns of antimicrobial resistant bacteria carriage in a nesting colony of Yellow‐legged Gulls from an industrialized area in Southern France. We found that carriage of antimicrobial resistant bacteria by the chicks increased over time and was not spatially structured within the gull colony. This study highlights the complexity of antimicrobial resistant bacteria carriage in a free‐ranging wildlife species and contributes to our understanding of the public health risks and implications associated with this carriage.

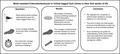

## 1 INTRODUCTION

Antimicrobial resistance (AMR), defined as the ability of a microorganism to resist a substance that would normally be inhibitory or lethal to it (WHO, 2020), is becoming one of the most serious modern threats to human health worldwide (Huemer et al., 2020; Morrison & Zembower, 2020). The causes of AMR are various, but the main ones clearly result from anthropogenic activities, especially antimicrobial use and misuse in human and veterinary medicine (Ayukekbong et al., 2017; Michael et al., 2014). Because of the close connections between human, animal, and environmental health, it is also increasingly recognized that a “One Health” approach is essential when addressing problems associated with AMR (Singh et al., 2021; Swift et al., 2019; White & Hughes, 2019).

Among the important topics that are still only partially elucidated is the role of wildlife in the epidemiology of antimicrobial‐resistant bacteria (hereafter AMRB). Numerous wild species, notably mammals and birds, have been shown to carry a large diversity of AMRB and associated AMR genetic determinants in their feces (Goulas et al., 2020; Ramey & Ahlstrom, 2020; Vittecoq et al., 2016). Wildlife do not normally have direct exposure to antibiotics, but they can be exposed through anthropogenic sources, such as food and water contaminated with pharmaceutical effluents and sewage (Al‐Bahry et al., 2009; Alroy & Ellis, 2011). Wildlife can also be exposed to antimicrobial‐resistant substances through the soil, and some of this exposure may be from natural sources (Cytryn, 2013; Nesme & Simonet, 2015). However, it is unclear whether wildlife are maintenance hosts or bridge hosts of antimicrobial resistance. Longitudinal and spatiotemporal studies are needed to address this question.

Gulls, especially species feeding from anthropogenic sources, are regularly reported to carry AMRB of clinical importance for human and livestock (Aberkane et al., 2017; Russo et al., 2021; Stedt et al., 2014; Vergara et al., 2017). As an example, landfill‐foraging gulls contribute to the maintenance of multiresistant *Escherichia coli* of clinical importance in Alaska (Ahlstrom et al., 2019), and it has been experimentally shown that they have the potential to act as bridge hosts for colistin‐resistant *E*. *coli* between the environment and humans/livestock (Franklin et al., 2020). The yellow‐legged gull is a common landfill‐foraging species that is widely distributed along Mediterranean coasts (Vidal et al., 1998). It mainly feeds from anthropogenic sources, including industrial fishing discards and human waste from landfills, and waste from other human activities (Duhem et al., 2003, 2008; Ramos et al., 2009). Depending on the context, these gulls can be considered either as maintenance hosts, contributing to the maintenance of AMRB in the environment, or as environmental reservoirs or bridge hosts, providing a link through which AMRB can be transmitted from the environment to humans or livestock (Caron et al., 2015; Franklin et al., 2020).

Our study was performed in a breeding colony of yellow‐legged gulls in the Rhone delta in the Camargue (southern France). Previous work by us on this colony found evidence for the occurrence of AMR enterobacteria in gull chicks nesting on this islet, for example, carbapenem‐resistant *E*. *coli* isolates positive for the *bla* VIM‐1 gene (Vittecoq et al., 2017). The objectives of this study included the use of a longitudinal study design to explore the temporal patterns of multiresistant Enterobacteriaceae isolates collected from yellow‐legged gull chicks during their first weeks of life, and to identify the genetic diversity and spatial structuring of third‐generation cephalosporin (3GC)‐resistant enterobacteria among chicks on the island. We focused our genetic analyses on 3GC‐resistant Enterobacteriaceae because they represent one of the most important public health threats associated with antimicrobial resistance in Europe (Rohde et al., 2018; Rottier et al., 2021), with 3GC‐resistant *E*. *coli* causing bloodstream infections that may increase hospital stays and even cause mortalities (de Kraker et al., 2011). Understanding these dynamics is important since it may help to identify high‐risk areas and contexts for AMRB transfer from the environment to humans or livestock as well as to develop efficient surveillance programs to monitor these transfer risks (Torres, Carvalho, et al., 2020).

## 2 MATERIALS AND METHODS

### 2.1 Study site and sampling data

**FIGURE 1:**
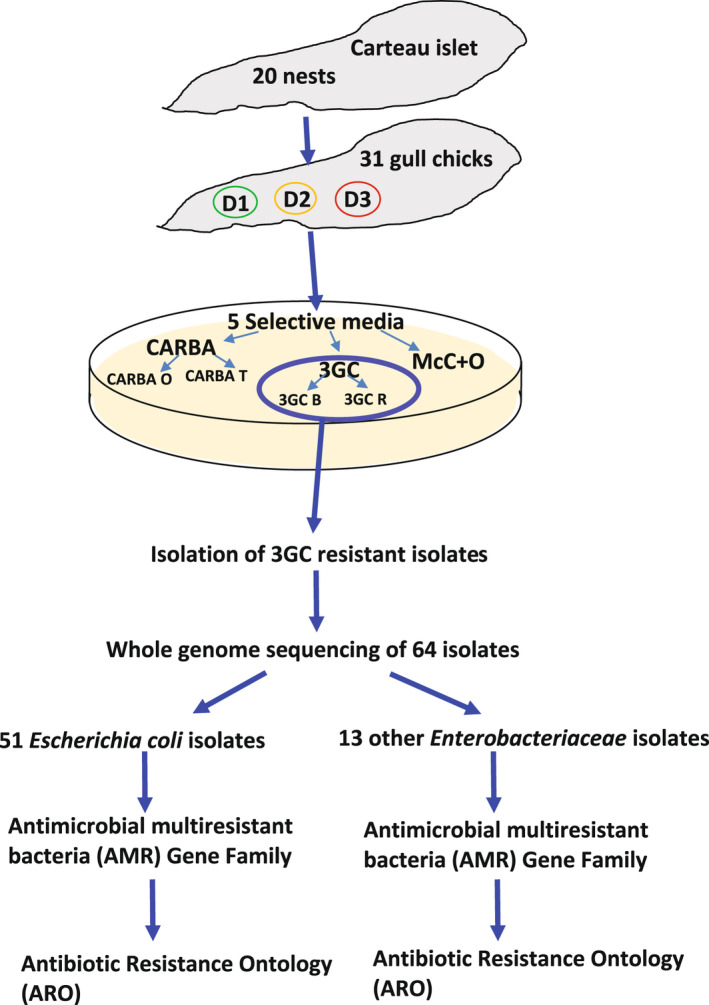
Diagram of analyses performed on 31 gull chicks sampled on Carteau islet. Sampling dates were D1: April 25, 2016, D2: May 5, 2016, D3: May 17, 2016. 3GC B & R: Extended‐spectrum beta‐lactamases; McC+O, MacConkey with Ofloxacin antibiotic; CARBA O, Carbapenemase‐producing *Enterobacteriaceae*, mainly KPC and metallo‐carbapenemase‐type CPE; CARBA T, Carbapenemase‐producing *Enterobacteriaceae* OXA‐48 type CPE; AMR, antimicrobiological‐resistant gene family; ARO, antibiotic resistance ontology determined according to protocols on the CARD website

The Rhone delta in the Camargue is a large biodiversity hotspot that hosts around 300 bird species annually. It also has industrial (e.g., petrochemical industry and metallurgy) and tourism activities that strongly impact the surrounding ecosystems (Fraixedas et al., 2019). This study was conducted on the small islet of Carteau located near the village of Port‐Saint‐Louis du Rhône (4°51′26.50″E, 43°22′39.93″N) in the Camargue area of southern France (Figure S1). The islet, which is approximately 210 m long and 65 m wide (total area 6800 m^2^), harbors a colony of yellow‐legged gulls (*Larus michahellis*) that has an estimated population of 400 breeding pairs. We sampled 31 gull chicks from 20 nests: 54 cloacal swabs were collected from chicks aged from 1 to 3 weeks on three dates in 2016 (25 April, 5 May, 17 May), hereafter denoted D1, D2, and D3. We sampled 23 individuals on two occasions (11 on D1 and D3, 12 on D2 and D3), and 8 chicks were only sampled once (five on D1 and three on D2). Figure 1 summarizes the schematic sequence of the protocols performed, from sampling to genomic analyses.

#### 2.1.1 Ethics statement

Our study was approved by the *Direction Départementale des Territoires et de la Mer*, Prefectural Order No. 13‐2016‐03‐14‐003 of March 14, 2016, granting authorization in Article L.411‐1, under Article L.411‐2, 4 of the Environmental Code, for sampling protected species as part of a research program on antibiotic‐resistant bacteria that are transmissible between humans and wildlife. Birds were handled and sampled under the supervision of two registered bird banders from the Museum National d’Histoire Naturelle, Paris (Thomas Blanchon and Antoine Arnaud). Permits for fieldwork were issued by the municipality of Port‐Saint‐Louis and the Communauté d’Agglomération Toulon Provence Méditerranée.

### 2.2 Isolation of bacteria

Immediately after sampling, the cloacal swabs were placed in Oxoid Tryptone Soya Broth (Thermo Scientific), transported in a cooler to the laboratory, and incubated at 37°C overnight. Following incubation, a loopful was streaked on agar plates with different media, as follows:

1. MacConkey without Cristal Violet (Bio‐Rad reference 3564154) agar supplemented with an Ofloxacin antibiotic (10 µg/ml) (McC+O, Sigma ref. O8757‐1G) for selective isolation of Enterobacteriaceae at broad‐spectrum, first‐generation bactericidal fluoroquinolone.
2. Selective chromogenic medium for the screening of carbapenemase‐producing Enterobacteriaceae, primarily KPC and metallo‐carbapenemase‐type CPE (CARBA O), and OXA‐48 type CPE (CARBA T) (bioMérieux ref. 414685).
3. 3GC agar for selective isolation medium (3GC B: Drigalski with Cefotaxime [15 µg/ml] and 3GC R: MacConkey with Ceftazidime [20 µg/ml]) (bioMérieux ref. AEB525770) for screening 3GC‐resistant Enterobacteriaceae. These two media select for 3GC‐resistant enterobacteria, hereafter referred to as 3GC B and 3GC R.

Bacteria were incubated for 24–48 h depending on the observation of bacterial colonies (presence [1]/absence [0]) on each medium (Table S1).

### 2.3 Whole‐genome sequencing

Whole‐genome DNA was isolated using the DNA blood and tissue kit (Qiagen) according to the manufacturer's instructions, and DNA concentration was estimated using a Qubit 2.0 fluorometer. Whole bacterial genome library preparation and sequencing into two NextSeq High‐Output multiplexed 2 × 150 bp paired‐end runs (Illumina) were performed by FISABIO Genomics Service. Bacterial isolates were specifically assigned according to the 16S rRNA sequences by FISABIO. Thirty‐six bacterial DNA extracts were of insufficient quality to be sequenced and were thus excluded from subsequent analyses. Thus, out of 100 3GC‐resistant isolates, the genomes of 64 3GC‐resistant bacterial isolates were completely sequenced and considered for further analyses (see detailed information on these isolates in Table S2). For the de novo genome assembly of each bacterial isolate, right‐tail quality trimming (with a minimum quality threshold of 20) was performed using the FASTX‐Toolkit v 0.0.14 (http://hannonlab.cshl.edu/fastx_toolkit/).

PRINSEQ v 0.20.4 (Schmieder & Edwards, 2011) was next used to remove reads containing undefined nucleotides (“N”), those shorter than 75 bps, and those left without a pair after the read cleaning process. The remaining reads were assembled using SPAdes v 3.10.1 (Schmieder & Edwards, 2011), which entailed performing read error correction and mismatch correction with k‐mer lengths of 33, 55, 77, and 99. From the resulting contigs, those that were shorter than 200 bp were dropped.

### 2.4 Resistome analysis

We refined the analysis by determining the list of AMR gene families and genes present per isolate. The Comprehensive Antibiotic Resistance Database (CARD) (Jia et al., 2017) was used to identify and analyze the antimicrobial‐resistant gene families and genes. A novel genome analysis tool, Resistance Gene Identifier application (RGI, version 5), was also used to identify antibiotic resistance genes. Antibiotic Resistance Ontology (ARO) is at the core of CARD; it is organized into six branches giving details on antimicrobial compounds, resistance genes and mutations, drug targets, and resistance mechanisms (see more details in Jia et al., 2017). The data analyzed per contig were detections of antimicrobial‐resistant gene families and ARO. Indeed, while investigating which resistance genes were present from each individual chick, we used information obtained from the ARO. In addition, to investigate differences between 3GC‐resistant isolates containing the same number of AMR gene families and AROs, all ARO sequences were aligned for comparison. This way we could determine whether all 3GC‐resistant isolates with the same numbers of AMR gene families and AROs were in fact different.

### 2.5 Statistical analysis

#### 2.5.1 Statistical analysis of bacteria grown on culture media

Using data from the isolates grown from the 54 swabs on each of the five different culture media, we tested the effect of chick age on the prevalence of bacteria resistant to each of the five selective media via generalized linear (binomial) mixed models. For each sample, bacteria were grown on N media, with N varying from 0 to 5. We studied the variable N according to the sampling date by means of Wilcoxon signed‐rank tests. We separately tested the differences between dates D1 and D3 and the differences between dates D2 and D3, since no individual chick was sampled on dates D1 and D2.

#### 2.5.2 Statistical analysis of the temporal patterns and spatial distribution of AMR gene diversity in gull chicks

We studied whether the presence of ARO and AMR tended to vary according to chick age using genetic data. For each isolate, we counted the number of resistance genes (ARO) and also the number of AMR gene families present with at least one gene in the isolate genome. We then compared values from the three sampling dates using Poisson generalized linear mixed models with chick as a random effect.

The spatial distribution of 3GC‐resistant Enterobacteriaceae isolated from chicks was determined as follows. First, we used a permutation test to determine whether isolates from the same bird (respectively nest) displayed closer resistance profiles than isolates from different birds (respectively nests). The distance between the repertoires of AMR genes in each pair of isolates was the Jaccard distance computed on the ARO profiles (including AMR genes and their mutations). Then, for each pair of nests *A* and *B*, we computed the geographic distance *d* (*A*, *B*) between them and the minimum Jaccard distance *g* (*A*, *B*) between each of the pairs of bacteria, one from nest *A* and the other from nest *B*. The correlation between distances *d* and *g* was assessed via a Mantel test. Details of the statistical procedures can be found in the Appendix S1. All computations were performed with R software (R Core Team, 2020).

## 3 RESULTS

### 3.1 Temporal patterns of multiresistant Enterobacteriaceae isolated on selective media

**FIGURE 2:**
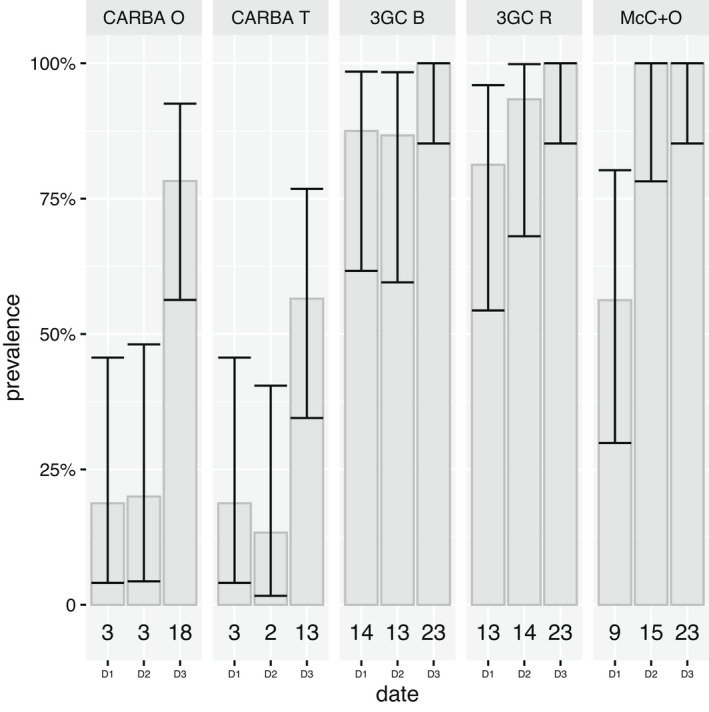
Absolute number (bottom) and corresponding prevalence in gull chicks for which any *Enterobacteriaceae* was isolated from the different selective media by sampling date (D1 to D3). The total number of chicks sampled in D1, D2, and D3 were 16, 15, and 23, respectively. Error bars are exact binomial 95% confidence intervals

**FIGURE 3:**
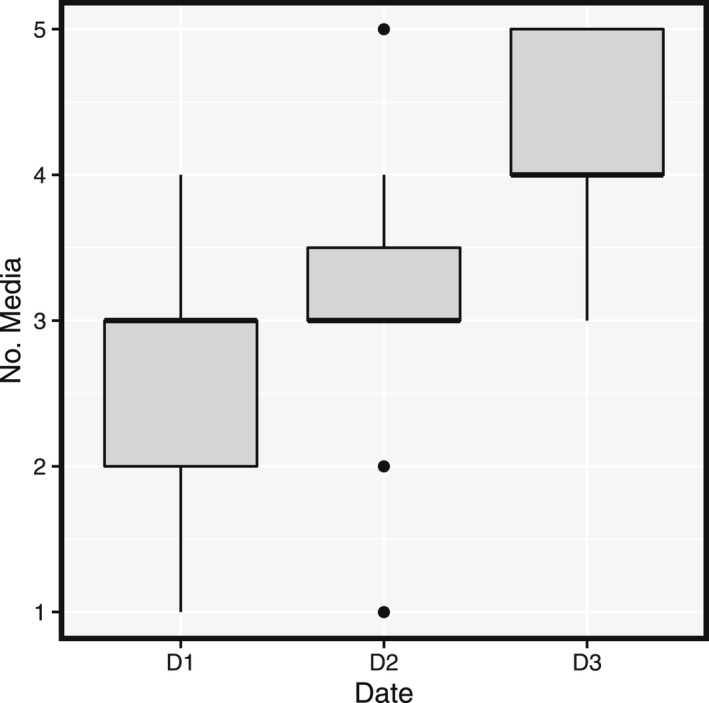
Box‐and‐whiskers plots of the number of selective media per swab for which any *Enterobacteriaceae* colony was detected, by sampling date (16 chicks sampled on D1, 15 on D2, and 23 on D3). D1: April 25, 2016; D2: May 5, 2016; D3: May 17, 2016. The bold line indicates the median, the box extends from the 1st to the 3rd quartile, and the whiskers extend to 1.5 times the interquartile width

We recovered 189 resistant bacterial isolates using the 5 selective solid media: 47 on MacConkey supplemented with Ofloxacin, 42 on CARBA O and T, and 100 on 3GC B and R media. The presence/absence of bacterial colony growth on each media for each swab collected is indicated in Table S1. The most common bacterial isolates for all three sampling dates were those resistant to 3GC (Table 1). The number of 3GC‐resistant enterobacteria isolates collected per gull chick varied from one to four (Figure 2). There was a significant increase with time in the proportion of resistant bacterial isolates for CARBA O (*p* ≤ .0001), CARBA T (*p* ≤ .0001), McConkey+O (*p* ≤ .0001), and 3GC R (*p* ≤ .0001). The increase was not statistically significant for 3GC B (*p* = .11). There was also an increase in the detection of resistant Enterobacteriaceae over time (Figure 3). For chicks sampled twice (*n* = 23), we found that the number of selective media in which resistant bacteria were able to grow increased with time (paired Wilcoxon signed‐rank test; *p* = .00098 for chicks sampled at D1 and D3, *p* = .0015 for chicks sampled at D2 and D3).

**TABLE 1:** Number of 3GC‐resistant *Enterobacteriaceae* species by sampling date in gull chicks

	*Cf*	*Ea*	*Ec*	*Encl*	*Ef*	*Ek*	*Ha*	*Ka*	*Kp*	*Pm*	Total
D1	0	0	9	0	0	1	0	0	0	0	10
D2	0	0	15	1	1	0	0	0	0	1	18
D3	1	1	27	0	0	0	1	1	4	1	36
Total	1	1	51	1	1	1	1	1	4	2	64

Note

Sampling dates D1: 25 April 2016, D2: 5 May 2016, D3: 17 May 2016.

Abbreviations: *Cf*: *Citrobacter freundii*; *Ea*: *Escherichia alberti*; *Ec*: *Escherichia coli*; *Encl*: *Enterobacter cloacae*; *Ef*: *Escherichia fergusonii*; *Ek*: *Enterobacter kobei*; *Ha*: *Hafnia alvei*; *Ka*: *Klebsiella aerogenes*; *Kp*: *Klebsiella pneumoniae*; *Pm*: *Proteus mirabilis*.

### 3.2 Genetic diversity, spatial and temporal structure of 3GC‐resistant Enterobacteriaceae in gull chicks

#### 3.2.1 Enterobacteriaceae species

Based on their 16S rRNA sequences, the 64 3GC‐resistant isolates sequenced belonged to one of ten species: *Citrobacter freundii* (1), *Enterobacter cloacae* (1), *Enterobacter kobei* (1), *Escherichia albertii* (1), *Escherichia coli* (51), *Escherichia fergusonii* (1), *Hafnia alvei* (1), *Klebsiella aerogenes* (1), *Klebsiella pneumonia* (4), *and Proteus mirabilis* (2). Details of the numbers of different enterobacteria species identified by sample date are shown in Table 1.

#### 3.2.2 Spatial and temporal genetic diversity of 3GC‐resistant isolates

**FIGURE 4:**
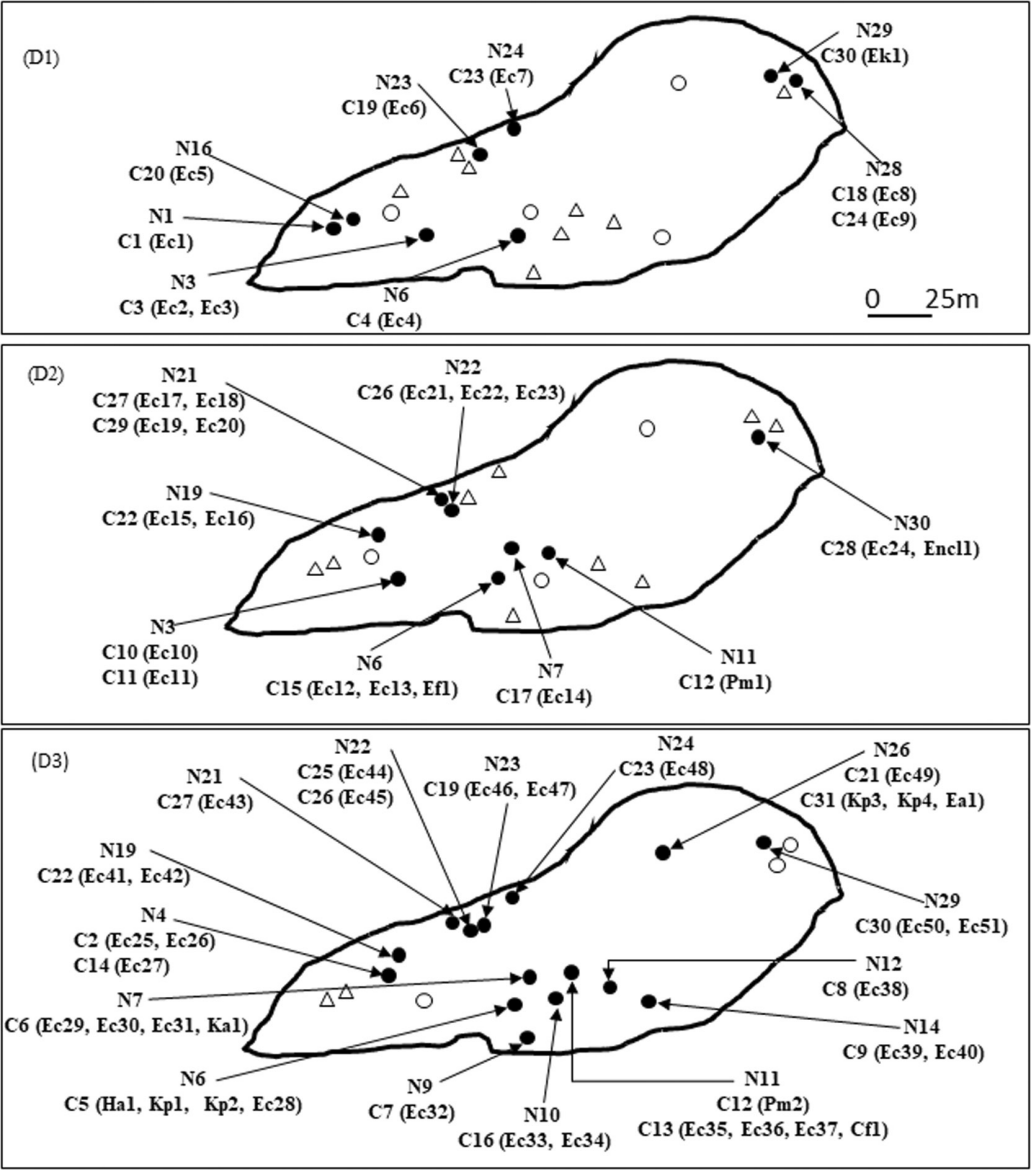
Location and distribution of *Enterobacteriaceae* isolates sampled from gull chicks per nest on Carteau islet. Only nests and chicks with *Enterobacteriaceae* isolates whose genomes were completely sequenced are indicated. D1, D2, and D3: sampling dates; N: nest number; C: gull chick number; black full circles: sampled chick with 3GC‐resistant isolates; black empty circles: sampled chick without 3GC‐resistant isolates; black empty triangles: non‐sampled chick. The *Enterobacteriaceae* species are indicated in parentheses: Cf, *Citrobacter freundii*; Ea, *Escherichia alberti*; Ec, *Escherichia coli*; Encl, *Enterobacter cloacae*; Ef, *Escherichia fergusonii*; Ek, *Enterobacter kobei*; Ha, *Hafnia alvei*; Ka, *Klebsiella aerogenes*; Kp, *Klebsiella pneumoniae*; Pm, *Proteus mirabilis*. The AMR and ARO abundances of each *Enterobacteriaceae* species are shown in Table S2

We sampled 20 nests with one to three gull chicks per nest. The numbers of bacterial‐resistant isolates detected over time were as follows: 10 3GC‐resistant bacterial isolates for 9 chicks from 8 nests on D1; 18 3GC‐resistant isolates for 10 chicks from 8 nests on D2; and 36 3GC‐resistant isolates for 19 chicks from 15 nests on D3 (linear trend test; number of isolates per bird, *p* = .22) (Figure 4).

The entire genome of 64 3GC‐resistant bacterial isolates from gull chicks were sequenced from 1027 million total reads (average of 16 million per isolate; range values are min = 4,310,105, max = 50,502,127). Using the CARD application, we could identify 135 different AROs belonging to 44 AMR gene families in the 64 bacterial isolates. The list of AROs detected for each identified gene family is presented in Table S3. The estimated prevalence of ARO in bacterial isolates from the 31 chicks varied between 0.03 and 1.00 (Table S4).

Among the resistance genes observed, those of clinical importance include the beta‐lactamase genes CTX‐M, SHV, TEM, and CMY. They were mostly carried by *E*. *coli* isolates (see prevalence in Table S4). We also identified two *P*. *mirabilis* carrying a CMY‐2 beta‐lactamase gene as well as another *P*. *mirabilis* isolate carrying both the CTX‐M 15 and TEM‐1 genes. Another subset of the 135 AROs observed were more common genes that are of less concern but that are not shared by most of the isolates, such as sulfonamide resistance genes (sul1, sul2, and sul3) or trimethoprim‐resistant dihydrofolate reductase genes (drf‐A1, A5, A1, and A17).

**FIGURE 5:**
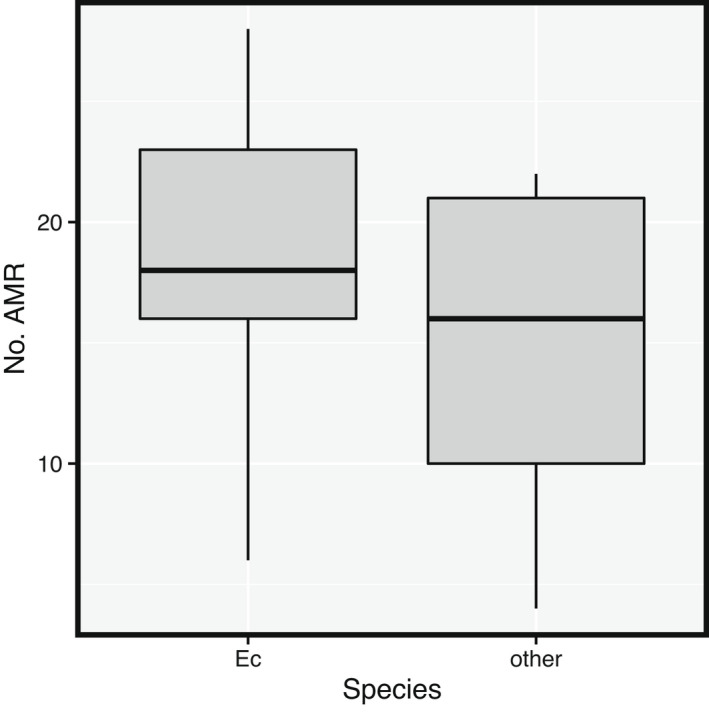
Box‐and‐whiskers plots of AMR gene families isolated from gull chicks. The AMR gene families shown have representatives in the complete genomes of *Escherichia coli* isolates (Ec) and other 3GC‐resistant *Enterobacteriaceae* species isolates pooled (other). The pooled 3GC‐resistant *Enterobacteriaceae* include *Citrobacter freundii*, *Escherichia alberti*, *Enterobacter cloacae*, *Escherichia fergusonii*, *Enterobacter kobei*, *Hafnia alvei*, *Klebsiella aerogenes*, *Klebsiella pneumoniae*, and *Proteus mirabilis*. See Figure 4 legend for further details

The number of AMR gene families was significantly greater in *E*. *coli* genomes than in other Enterobacteriaceae species (Poisson GLMM, *p* = .0013) (Figure 5). If we consider the distribution of AMR abundance by date, sampling date were not significantly associated with the number of AMR gene families (Poisson GLMM, *p* = .23). More specifically, *E*. *coli* was the only species present on all three sampling dates (median numbers of AMR for the three dates = 18, 19, and 18) while other species were scarce.

As observed for AMR gene families, *E*. *coli* isolates carried significantly more AROs than other isolates (Poisson GLMM, *p* ≤ .0001). Considering the number of ARO per isolate by sampling date, there was a significant positive linear trend over time (Poisson GLMM, *p* = .0026) (Figures S2 and S3). All 3GC‐resistant isolates with the same numbers of AMR gene families and AROs were different except for isolates Ec39 (D3, nest 14) and Ec51 (D3, nest 29), which were genetically identical in all ARO sequences.

**FIGURE 6:**
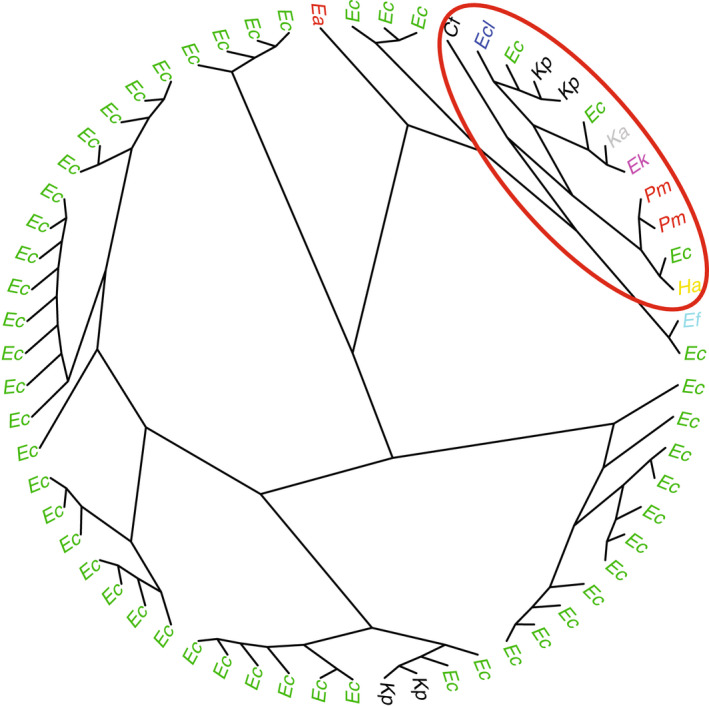
Classification tree based on the number of discrepancies in the presence/absence of AROs. The red ellipse shows a cluster that includes most isolates from species other than *Escherichia coli*. *Cf*, *Citrobacter freundii*; *Ea*, *Escherichia alberti*; *Ec*, *Escherichia coli*; *Encl*, *Enterobacter cloacae*; *Ef*, *Escherichia fergusonii*; *Ek*, *Enterobacter kobei*; *Ha*, *Hafnia alvei*; *Ka*, *Klebsiella aerogenes*; *Kp*, *Klebsiella pneumoniae*; *Pm*, *Proteus mirabilis*

The Jaccard distances between AROs in two isolates from the same nest were not significantly smaller than the distances between two isolates from different nests (permutation test, one‐sided alternative, *p* = .53). The same conclusion was reached when comparing isolates from the same bird versus isolates from different birds (*p* = .17). Jaccard distances between pairs of Enterobacteriaceae isolates were used to compute a neighbor‐joining classification tree. This classification tree shows that most species other than *E*. *coli* were clustered into two close nodes (red ellipse in Figure 6). Finally, there was no correlation between geographical distances between nests and distances based on the presence or absence of ARO genes (Mantel permutation test, 500 simulations, *p* = .14).

## 4 DISCUSSION

Ramey and Ahlstrom (2020) encouraged the study of bacteria carried by synurbic gulls that should include a detailed search for the presence of resistance genes combined with precise data on the bird's ecology. Using this approach, our study revealed the wide diversity of resistance genes present in bacteria carried by yellow‐legged gull chicks. An important originality of this work is the implementation of three sampling periods, which allowed us to follow the temporal progression of resistant isolates. Moreover, monitoring chicks by nest brought us spatial information that is usually lacking in studies focusing on AMR in wildlife, especially those conducted on gulls.

One important result of this study is the clear increase in the diversity of resistant enterobacteria over time between chick hatching and fledging. This phenomenon was accompanied by an increase in the proportion of individuals carrying AMRB. Thus, carrying is not constant in the population, but highly dependent on the species’ phenology. From these findings, one might hypothesize either that AMR are rapidly and positively selected in chicks and/or that AMR accumulate over time. This could occur because the parents’ food supply changes with chick age (Ramos et al., 2009) and/or because parents, through time, are more likely to have foraged in different areas (Méndez et al., 2020; Ramos et al., 2009). Another possible contributing factor could be the increasing number of exchanges with other chicks, since older individuals frequently leave the nest (Martínez‐Abraín et al., 2003; Tinbergen, 1989). These frequent exchanges combined with shared sources of food from the parents could contribute to explaining the lack of spatial structure that we observed. Considering our relatively small sample size (54 cloacal swabs collected from 31 chicks), it would be interesting to extend this study to other landfill‐foraging gull colonies to see if the same patterns are observed.

These findings also highlight the importance of considering inter‐individual differences when sampling wildlife, notably related to age. In our case, if one wishes to highlight the diversity of the resistome carried by the studied population, the oldest chicks will be more representative. In situations like this, where the capture of adults is difficult, another way to investigate resistomes would be to collect droppings. We have not used this method because the number of individuals being considered would remain unknown, and thus the proportion of birds carrying the bacteria of interest would also be unknown. However, it would be interesting to compare the diversity of resistomes observed using the two methods. Indeed, if the species is to be studied as a sentinel of environmental contamination and used to document sources of this contamination, the two ways of obtaining samples could be complementary.

Another important finding of this study is the evidence that gull chicks carry multiresistant enterobacteria that can pose major clinical issues. We notably detected *E*. *coli* isolates carrying various genes associated with β‐lactamase production, including 10 different genes of the CTX‐M family that currently present major therapeutic and infection control challenges worldwide (Critchley et al., 2019; Devi et al., 2020; Livermore et al., 2007). It should be noted that this observed resistome only covers the enterobacteria that were selectively cultured on our media. Because there are so many studies focusing on *E*. *coli* isolates with antimicrobial‐resistant genes, a core resistome shared by most (95%) *E*. *coli* isolates was identified by Goldstone and Smith (2017). In our study, this core resistome represents 27 of the AROs observed in *E*. *coli*. Future studies using a high‐throughput sequencing approach could allow further exploration of this resistome. However, it is difficult at this stage to determine the consequences of this percentage in terms of public and veterinary health threats. More work is required to effectively use yellow‐legged gulls—or other landfill‐foraging gull species—as sentinels for antimicrobial resistance. First, better knowledge of gull movements, movement variability, and determinants of movements is needed. The growing use of GPS‐tracking will allow increasingly detailed data on these movements that will help to evaluate the risks posed by gulls carrying AMRB (e.g., Navarro et al., 2019).

The presence of 3GC‐resistant enterobacteria including *Escherichia coli*, *Proteus mirabilis*, *Klebsiella pneumonia*, and *Enterobacter cloacae* isolates has already been observed in this gull colony (Aberkane et al., 2017; Bonnedahl et al., 2009; Vittecoq et al., 2017), so it seems that the presence of 3GC‐resistant bacteria is the result of regular contamination rather than a one‐time event. An alternative explanation could be that 3GC bacteria have become part of the yellow‐legged gull's common resistome, and these birds are now a maintenance host. A growing number of studies have shown that synurbic gulls are very frequent carriers of AMRB, including 3GC‐resistant enterobacteria (Dolejska et al., 2016; Hernandez et al., 2013; Vergara et al., 2017). It has thus been proposed that synurbic gulls could be valuable sentinels of environmental AMRB and by extension of the environmental resistome (Ramey & Ahlstrom, 2020).

### 4.1 Concluding remarks

Over the last decade, wild species have been identified that could be efficient sentinels of environmental contamination (Ramey & Ahlstrom, 2020; Torres, Fernandes, et al., 2020). Landfill‐foraging gulls, such as the yellow‐legged gull, are among these sentinels. To use sentinels at their maximum potential—to identify where and how exchanges of AMRB occur among the environment, humans, and livestock, precise information concerning the wild individuals sampled and their use of the environment must be combined with genetic data to characterize the resistant bacteria they carry. Our study can be seen as a step toward the implementation of this approach, which, over the long term and by allowing comparisons between different geographical areas, will contribute to controlling the spread of antimicrobial resistance. In addition, sampling potential sources of gull exposure to AMRB at different periods of the year (e.g., water surrounding the colonies or foraging areas, food sources) would allow a better understanding of how exchanges take place. Comparing AMRB and AMR genes carried by wildlife with those found in humans, livestock, and the environment using a “One Health” approach and including data on the spatiotemporal dynamics of the carrying patterns will be of great interest in the future.

## AUTHOR CONTRIBUTIONS

**Marion Vittecoq:** Conceptualization (equal); Funding acquisition (equal); Investigation (equal); Writing – original draft (lead); Writing – review & editing (lead). **Lionel Brazier:** Conceptualization (equal); Data curation (equal); Formal analysis (equal); Investigation (equal); Visualization (equal); Writing – review & editing (equal). **Eric Elguero:** Formal analysis (equal); Visualization (equal); Writing – review & editing (equal). **Ignacio Bravo:** Data curation (equal); Formal analysis (equal); Writing – review & editing (equal). **Nicolas Renaud:** Conceptualization (equal); Investigation (equal); Writing – review & editing (equal). **Alejandro Manzano‐Marín:** Data curation (equal); Investigation (equal); Writing – review & editing (equal). **Franck Prugnolle:** Writing – review & editing (equal). **Sylvain Godreuil:** Writing – review & editing (equal). **Thomas Blanchon:** Conceptualization (equal); Investigation (equal); Writing – review & editing (equal). **François Roux:** Conceptualization (equal); Investigation (equal); Writing – review & editing (equal). **Patrick Durand:** Conceptualization (equal); Data curation (equal); Formal analysis (equal); Investigation (equal); Visualization (equal); Writing – review & editing (equal). **François Renaud:** Conceptualization (equal); Funding acquisition (equal); Investigation (equal); Writing – review & editing (equal). **Frédéric Thomas:** Writing – review & editing (equal).

## CONFLICT OF INTEREST

The authors declare that they have no conflicts of interest to report.

## Supporting information

AppendixS1

## Data Availability

Metadata and NGS sequences have been deposited in the National Center for Biotechnology Information (NCBI) under the BioProject accession number PRJNA705836 and are available from https://www.ncbi.nlm.nih.gov/sra. Data are available at Dryad https://doi.org/10.5061/dryad.fxpnvx0vf.
