# Supplementary material for: Multiresistant Enterobacteriaceae in yellow‐legged gull chicks in their first weeks of life
Source: Ecol Evol. 2022 Jun 11;12(6):e8974. doi: 10.1002/ece3.8974 (PMC9188031; doi:10.1002/ece3.8974)
Supplement: Supplementary file 1 — AppendixS1 [file ECE3-12-e8974-s001.docx]

**Appendix**

**Statistical analyses**

We detail here the statistical method by which we tested whether two isolates from the same nest, be they from the same chick or not, are closer to each other than two isolates from two different nests. The same approach was used to study the closeness of isolates from the same chick vs isolates from two different chicks and will not be detailed here. In this analysis, closeness is taken as the Jaccard distance between two profiles of presence/absence of the 135 resistance genes.

The 64 isolates were collected in 20 nests, four of them having yielded a single isolate. These four nests and corresponding isolates were removed from the analysis. We then computed the Jaccard distances between each pair of different isolates. The 1770 distances were divided in two groups: one group called *intranest* of 105 distances between two isolates coming from the same nest, and the other group called *internest* of 1665 distances between two isolates coming from two different nests. If there was some degree of clustering of the isolates inside the nests, one expects that the average intranest distance D_intra_ were lesser than the average inter-nest distance D_inter_. Hence the difference D = D_inter_ – D_intra_ was computed, with the idea that a large positive value is indicative of clustering.

We then assigned the 60 isolates to the 16 nests at random, but keeping the numbers of isolates per nest as they were in the dataset. Under this permutation, we computed a value D* = D*_inter_ – D*_intra_. This process was repeated 10,000 times. Under the null hypothesis that there is no clustering, the observed value would be indistinguishable from any one of the simulated values, whereas in the alternative hypothesis it would be in the upper tail of the distribution of simulated values. Hence the P-value was taken as the proportion of D* that are larger than D.


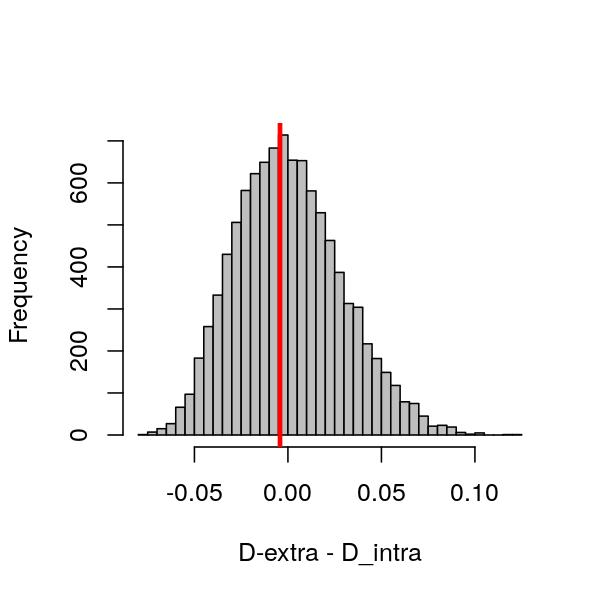
The figures shows the distribution of the 10,000 simulated values of D*_inter_ – D*_intra_ , the red line indicates the position of the observed difference D_inter_ – D_intra_.

**Appendix Table 1.** Collecting data from gull chicks with the presence (1) or absence (0) of bacterial colonies on different media at the three sampling dates. The last 3 columns indicate the number of media on which some bacteria from the given sample grew, Di corresponds to the sampling date D1 to D3. The average in the last row. Date D1 excludes the first 5 samples. *McC+O MacConckey with Ofloxacin antibiotic, ESBL B: Extended-Spectrum Beta-Lactamases B, ESBL R: Extended-Spectrum Beta-Lactamases R, Carba O: Carbapenemase-Producing Enterobacteriaceae O, Carb T: Carbapenemase-Producing Enterobacteriaceae T.*

|  |  | D1 |  |  |  |  | D2 |  |  |  |  | D3 |  |  |  |  |  |  |  |
| --- | --- | --- | --- | --- | --- | --- | --- | --- | --- | --- | --- | --- | --- | --- | --- | --- | --- | --- | --- |
| Nest | Chick | McC +O | 3GC B | 3GC R | CARB O | CARB T | McC +O | 3GC B | 3GC R | CARB O | CARB T | McC +O | 3GC B | 3GC R | CARB O | CARB T | **D1** | **D2** | **D3** |
| 1 | 1 | 1 | 1 | 1 | 0 | 0 |  |  |  |  |  |  |  |  |  |  | 3 |  |  |
| 3 | 3 | 1 | 1 | 1 | 0 | 0 |  |  |  |  |  |  |  |  |  |  | 3 |  |  |
| 3 | 12 |  |  |  |  |  | 1 | 1 | 1 | 0 | 0 |  |  |  |  |  |  | 3 |  |
| 3 | 13 |  |  |  |  |  | 1 | 1 | 1 | 0 | 0 | 1 | 1 | 1 | 1 | 1 |  | 3 | 5 |
| 4 | 2 | 0 | 1 | 1 | 0 | 0 |  |  |  |  |  | 1 | 1 | 1 | 0 | 0 | 2 |  | 3 |
| 4 | 16 |  |  |  |  |  | 1 | 0 | 0 | 0 | 0 | 1 | 1 | 1 | 0 | 1 |  | 1 | 4 |
| 6 | 4 | 0 | 1 | 1 | 0 | 0 |  |  |  |  |  |  |  |  |  |  | 2 |  |  |
| 6 | 5 | 1 | 1 | 1 | 0 | 0 |  |  |  |  |  | 1 | 1 | 1 | 1 | 0 | 3 |  | 4 |
| 6 | 19 |  |  |  |  |  | 1 | 1 | 1 | 1 | 0 |  |  |  |  |  |  | 4 |  |
| 7 | 26 | 1 | 0 | 0 | 0 | 0 |  |  |  |  |  | 1 | 1 | 1 | 0 | 1 | 1 |  | 4 |
| 7 | 29 |  |  |  |  |  | 1 | 1 | 1 | 0 | 0 |  |  |  |  |  |  | 3 |  |
| 9 | 7 | 1 | 0 | 0 | 0 | 0 |  |  |  |  |  | 1 | 1 | 1 | 1 | 0 | 1 |  | 4 |
| 10 | 21 |  |  |  |  |  | 1 | 0 | 1 | 0 | 0 | 1 | 1 | 1 | 0 | 0 |  | 2 | 3 |
| 11 | 14 |  |  |  |  |  | 1 | 1 | 1 | 0 | 1 | 1 | 1 | 1 | 1 | 1 |  | 4 | 5 |
| 11 | 15 |  |  |  |  |  | 1 | 1 | 1 | 1 | 1 | 1 | 1 | 1 | 1 | 1 |  | 5 | 5 |
| 12 | 8 | 0 | 1 | 1 | 1 | 1 |  |  |  |  |  | 1 | 1 | 1 | 1 | 1 | 4 |  | 5 |
| 14 | 9 | 0 | 1 | 0 | 1 | 1 |  |  |  |  |  | 1 | 1 | 1 | 0 | 1 | 3 |  | 4 |
| 16 | 33 | 0 | 1 | 1 | 0 | 1 |  |  |  |  |  |  |  |  |  |  | 3 |  |  |
| 19 | 35 |  |  |  |  |  | 1 | 1 | 1 | 1 | 0 | 1 | 1 | 1 | 1 | 0 |  | 4 | 4 |
| 21 | 47 |  |  |  |  |  | 1 | 1 | 1 | 0 | 0 | 1 | 1 | 1 | 1 | 1 |  | 3 | 5 |
| 21 | 49 |  |  |  |  |  | 1 | 1 | 1 | 0 | 0 | 1 | 1 | 1 | 1 | 0 |  | 3 | 4 |
| 22 | 40 |  |  |  |  |  | 1 | 1 | 1 | 0 | 0 | 1 | 1 | 1 | 1 | 1 |  | 3 | 5 |
| 22 | 44 |  |  |  |  |  | 1 | 1 | 1 | 0 | 0 | 1 | 1 | 1 | 1 | 0 |  | 3 | 4 |
| 23 | 32 | 1 | 1 | 1 | 0 | 0 |  |  |  |  |  | 1 | 1 | 1 | 1 | 0 | 3 |  | 4 |
| 24 | 36 | 1 | 1 | 1 | 0 | 0 |  |  |  |  |  | 1 | 1 | 1 | 1 | 0 | 3 |  | 4 |
| 26 | 34 | 0 | 1 | 1 | 0 | 0 |  |  |  |  |  | 1 | 1 | 1 | 1 | 1 | 2 |  | 5 |
| 26 | 56 |  |  |  |  |  | 1 | 1 | 1 | 0 | 0 | 1 | 1 | 1 | 1 | 0 |  | 3 | 4 |
| 28 | 31 | 1 | 1 | 1 | 0 | 0 |  |  |  |  |  |  |  |  |  |  | 3 |  |  |
| 28 | 38 | 1 | 1 | 1 | 1 | 0 |  |  |  |  |  | 1 | 1 | 1 | 1 | 1 | 4 |  | 5 |
| 29 | 50 | 0 | 1 | 1 | 0 | 0 |  |  |  |  |  | 1 | 1 | 1 | 1 | 1 | 2 |  | 5 |
| 30 | 48 |  |  |  |  |  | 1 | 1 | 1 | 0 | 0 | 1 | 1 | 1 | 1 | 1 |  | 3 | 5 |
| Prevalence | | 0.56 | 0.88 | 0.81 | 0.19 | 0.19 | 1.00 | 0.87 | 0.93 | 0.20 | 0.13 | 1.00 | 1.00 | 1.00 | 0.78 | 0.56 |  |  |  |
| Average |  |  |  |  |  |  |  |  |  |  |  |  |  |  |  |  | 2.63 | 3.13 | 4.35 |

**Appendix Table 2.** Distribution of the 64 3GC isolates detected in chicks per nest and per sampling date. Sampling dates D1: 4/25/2016, D2:5/2/2016, D3: 5/17/2016. *Enterobacteriaceae* species: Cf: *Citrobacter freundii;* Ea: *Escherichia alberti;* Ec*: Escherichia coli;* Encl: *Enterobacter cloacae;* Ef*: Escherichia fergusonii*; Ek: *Enterobacter kobei;* Ha*: Hafnia alvei;* Ka: *Klebsiella aerogenes;* Kp: *Klebsiella pneumoniae;* Pm: *Proteus mirabilis.* Number of AMR and ARO of each *Enterobacteriaceae* species are also shown in relation to the Figure 4.

| **Gull chick** | **Nest** | **Number of 3GC isolates per chick** | **Code of *Enterobacteriaceae* species** | **Number of AMR** | **Number of ARO** | **Sampling date** |
| --- | --- | --- | --- | --- | --- | --- |
| 1 | 1 | 1 | Ec1 | 12 | 50 | D1 |
| 2 | 4 | 2 | Ec25 | 26 | 66 | D3 |
| 2 | 4 |  | Ec26 | 17 | 58 | D3 |
| 3 | 3 | 2 | Ec2 | 14 | 40 | D1 |
| 3 | 3 |  | Ec3 | 18 | 58 | D1 |
| 4 | 6 | 1 | Ec4 | 20 | 58 | D1 |
| 5 | 6 | 4 | Ec28 | 15 | 24 | D3 |
| 5 | 6 |  | Ha1 | 4 | 5 | D3 |
| 5 | 6 |  | Kp1 | 22 | 64 | D3 |
| 5 | 6 |  | Kp2 | 22 | 62 | D3 |
| 6 | 7 | 4 | Ec29 | 18 | 28 | D3 |
| 6 | 7 |  | Ec30 | 17 | 58 | D3 |
| 6 | 7 |  | Ec31 | 16 | 54 | D3 |
| 6 | 7 |  | Ka1 | 7 | 16 | D3 |
| 7 | 9 | 1 | Ec32 | 21 | 65 | D3 |
| 8 | 12 | 1 | Ec38 | 23 | 67 | D3 |
| 9 | 14 | 2 | Ec39 | 17 | 55 | D3 |
| 9 | 14 |  | Ec40 | 23 | 64 | D3 |
| 10 | 3 | 1 | Ec10 | 17 | 56 | D2 |
| 11 | 3 | 1 | Ec11 | 17 | 55 | D2 |
| 12 | 11 | 2 | Pm1 | 10 | 11 | D2 |
| 12 | 11 |  | Pm2 | 14 | 23 | D3 |
| 13 | 11 | 4 | Cf1 | 12 | 24 | D3 |
| 13 | 11 |  | Ec35 | 28 | 73 | D3 |
| 13 | 11 |  | Ec36 | 17 | 39 | D3 |
| 13 | 11 |  | Ec37 | 14 | 53 | D3 |
| 14 | 4 | 1 | Ec27 | 26 | 66 | D3 |
| 15 | 6 | 3 | Ec12 | 14 | 49 | D2 |
| 15 | 6 |  | Ec13 | 16 | 54 | D2 |
| 15 | 6 |  | Ef1 | 19 | 40 | D2 |
| 16 | 10 | 2 | Ec33 | 14 | 53 | D3 |
| 16 | 10 |  | Ec34 | 23 | 64 | D3 |
| 17 | 7 | 1 | Ec14 | 25 | 67 | D2 |
| 18 | 28 | 1 | Ec8 | 20 | 60 | D1 |
| 19 | 23 | 3 | Ec6 | 14 | 53 | D1 |
| 19 | 23 |  | Ec46 | 17 | 57 | D3 |
| 19 | 23 |  | Ec47 | 17 | 55 | D3 |
| 20 | 16 | 1 | Ec5 | 17 | 55 | D1 |
| 21 | 26 | 1 | Ec49 | 25 | 67 | D3 |
| 22 | 19 | 4 | Ec15 | 26 | 70 | D2 |
| 22 | 19 |  | Ec16 | 26 | 69 | D2 |
| 22 | 19 |  | Ec41 | 6 | 4 | D3 |
| 22 | 19 |  | Ec42 | 12 | 18 | D3 |
| 23 | 24 | 2 | Ec7 | 23 | 65 | D1 |
| 23 | 24 |  | Ec48 | 16 | 54 | D3 |
| 24 | 28 | 1 | Ec9 | 20 | 60 | D1 |
| 25 | 22 | 1 | Ec44 | 26 | 67 | D3 |
| 26 | 22 | 4 | Ec21 | 23 | 69 | D2 |
| 26 | 22 |  | Ec22 | 19 | 62 | D2 |
| 26 | 22 |  | Ec23 | 17 | 38 | D2 |
| 26 | 22 |  | Ec45 | 24 | 70 | D3 |
| 27 | 21 | 3 | Ec17 | 25 | 69 | D2 |
| 27 | 21 |  | Ec18 | 26 | 71 | D2 |
| 27 | 21 |  | Ec43 | 19 | 59 | D3 |
| 28 | 30 | 2 | Ec24 | 22 | 61 | D2 |
| 28 | 30 |  | Encl1 | 22 | 32 | D2 |
| 29 | 21 | 2 | Ec19 | 15 | 53 | D2 |
| 29 | 21 |  | Ec20 | 14 | 52 | D2 |
| 30 | 29 | 3 | Ek1 | 9 | 18 | D1 |
| 30 | 29 |  | Ec50 | 25 | 66 | D3 |
| 30 | 29 |  | Ec51 | 23 | 64 | D3 |
| 31 | 26 | 3 | Ea1 | 16 | 46 | D3 |
| 31 | 26 |  | Kp3 | 19 | 30 | D3 |
| 31 | 26 |  | Kp4 | 21 | 31 | D3 |

**Appendix Table 3.** List of Antimicrobial resistance (AMR) gene families and Antibiotic Resistance Ontology (ARO) detected in the 64 *Enterobacteriaceae* strains sampled in 31 gull chicks.

| Code of AMR gene family | AMR Gene Family | ARO |
| --- | --- | --- |
| 1 | AAC(3) | AAC(3)-IIb, AAC(3)-VIa |
| 2 | AAC(6') | AAC(6')-Ib-cr |
| 3 | ABC-F ATP-binding cassette ribosomal protection protein | vgaC |
| 4 | ACC beta-lactamase | ACC-2 |
| 5 | ACT beta-lactamase | ACT-10, ACT-9 |
| 6 | ampC-type beta-lactamase | Escherichia coli ampC beta-lactamase |
| 7 | ANT(2'') | ANT(2'')-Ia |
| 8 | ANT(3'') | aadA, aadA13, aadA15, aadA17, aadA2, aadA21, aadA24, aadA5, aadA9 |
| 9 | antibiotic resistant nfsA | Escherichia coli nfsA mutations conferring resistance to nitrofurantoin |
| 10 | APH(3') | APH(3')-Ia |
| 11 | APH(3'') | APH(3'')-Ib |
| 12 | APH(6) | APH(6)-Id |
| 13 | ATP-binding cassette (ABC) antibiotic efflux pump | msbA, patA, YojI, Escherichia coli soxR with mutation conferring antibiotic resistance, TolC, Escherichia coli soxS with mutation conferring antibiotic resistance |
| 14 | chloramphenicol acetyltransferase (CAT) | catB3, catI, Shigella flexneri chloramphenicol acetyltransferase |
| 15 | CMY beta-lactamase | CMY-2, CMY-43 |
| 16 | CTX-M beta-lactamase | CTX-M-1, CTX-M-28, CTX-M-117, CTX-M-14, CTX-M-15, CTX-M-27, CTX-M-3, CTX-M-55, CTX-M-60, CTX-M-79 |
| 17 | cya adenylate cyclase | Escherichia coli CyaA with mutation conferring resistance to fosfomycin |
| 18 | elfamycin resistant EF-Tu | Escherichia coli EF-Tu mutants conferring resistance to Pulvomycin |
| 19 | fluoroquinolone resistant gyrA | Escherichia coli gyrA conferring resistance to fluoroquinolones, Salmonella enterica gyrA conferring resistance to fluoroquinolones |
| 20 | fluoroquinolone resistant gyrB | Morganella morganii gyrB conferring resistance to fluoroquinolone |
| 21 | fluoroquinolone self resistant parC | Escherichia coli parC conferring resistance to fluoroquinolone |
| 22 | fosfomycin thiol transferase | FosA3, FosA4 |
| 23 | General Bacterial Porin with reduced permeability to beta-lactams | Klebsiella pneumoniae OmpK37, Escherichia coli soxS with mutation conferring antibiotic resistance, marA, ramA |
| 24 | resistance-nodulation-cell division (RND) antibiotic efflux pump | Escherichia coli soxS with mutation conferring antibiotic resistance, evgA, evgS, H-NS, AcrB, AcrD, AcrE, AcrF, AcrS, adeF, baeR, baeS, cpxA, CRP, Enterobacter cloacae acrA, Escherichia coli acrA, Escherichia coli acrR with mutation conferring multidrug antibiotic resistance, Escherichia coli marR mutant conferring antibiotic resistance, gadW, gadX, Klebsiella pneumoniae acrA, mdtA, mdtB, mdtC, mdtE, mdtF, oqxA, marA, ramA, Escherichia coli soxR with muepting antibiotic resistance, TolC |
| 25 | major facilitator superfamily (MFS) antibiotic efflux pump | Escherichia coli soxS with mutation conferring antibiotic resistance, A, emrB, emrK, emrR, emrY, Escherichia coli mdfA, floR, mdtG, mdtH, mdtM, mdtN, mdtO, mdtP, mef(B), Salmonella enterica cmlA, tet(B), tet(C), tet(D), tet(J), tet(R), evgA, evgS, H-NS, Escherichia coli soxR with mutation conferring antibiotic resistance, TolC |
| 26 | GlpT | Escherichia coli GlpT with mutation conferring resistance to fosfomycin |
| 27 | kdpDE | kdpE |
| 28 | lincosamide nucleotidyltransferase (LNU) | linG |
| 29 | macrolide phosphotransferase (MPH) | mphA, Mrx |
| *30* | OXA beta-lactamase | OXA-1 |
| 31 | OXY beta-lactamase | OXY-4-1 |
| 32 | Penicillin-binding protein mutations conferring resistance to beta-lactam antibiotics | Haemophilus influenzae PBP3 conferring resistance to beta-lactam antibiotics |
| 33 | pmr phosphoethanolamine transferase | eptA, PmrF, ugd |
| 34 | PtsI phosphotransferase | Escherichia coli PtsI with mutation conferring resistance to fosfomycin |
| 35 | quinolone resistance protein (qnr) | QnrB1, QnrS1 |
| 36 | SHV beta-lactamase | SHV-32, SHV-76, SHV-83, SHV-178 |
| 37 | small multidrug resistance (SMR) antibiotic efflux pump | Escherichia coli emrE, qacH |
| 38 | streptothricin acetyltransferase (SAT) | SAT-2 |
| 39 | sulfonamide resistant sul | sul1, sul2, sul3 |
| 40 | TEM beta-lactamase | TEM-1, TEM-153, TEM-156 |
| 41 | trimethoprim resistant dihydrofolate reductase dfr | dfrA1, dfrA5, dfrA14, dfrA17 |
| 42 | UhpT | Escherichia coli UhpT with mutation conferring resistance to fosfomycin |
| 43 | undecaprenyl pyrophosphate related proteins | bacA |
| 44 | TRU beta-lactamase | TRU-1 |

**Appendix Table 4.** Prevalence of the 135 ARO detected in all bacterial isolates (N=64) and for *E.coli* (N=51) of the thirty-one gull chicks sampled. NC: number of chicks.

| List of ARO | NC | Prevalence (N=64) | Prevalence for *E.coli* (N=51) |
| --- | --- | --- | --- |
| AAC(3)-IIb | 7 | 0.23 | 0.10 |
| AAC(3)-VIa | 1 | 0.03 | 0.02 |
| AAC(6')-Ib-cr | 5 | 0.16 | 0.06 |
| aadA | 6 | 0.19 | 0.14 |
| aadA13 | 5 | 0.16 | 0.12 |
| aadA15 | 1 | 0.03 | 0.02 |
| aadA17 | 1 | 0.03 | 0.04 |
| aadA2 | 7 | 0.23 | 0.14 |
| aadA21 | 1 | 0.03 | 0.02 |
| aadA24 | 1 | 0.03 | 0.02 |
| aadA5 | 15 | 0.48 | 0.41 |
| aadA9 | 3 | 0.1 | 0.08 |
| ACC-2 | 1 | 0.03 | 0.00 |
| acrB | 30 | 0.97 | 0.90 |
| acrD | 6 | 0.19 | 0.18 |
| AcrE | 30 | 0.97 | 0.86 |
| AcrF | 30 | 0.97 | 0.86 |
| AcrS | 29 | 0.94 | 0.86 |
| ACT-10 | 1 | 0.03 | 0.00 |
| ACT-9 | 1 | 0.03 | 0.00 |
| adeF | 28 | 0.9 | 0.71 |
| ANT(2'')-Ia | 1 | 0.03 | 0.02 |
| APH(3')-Ia | 9 | 0.29 | 0.20 |
| APH(3'')-Ib | 21 | 0.68 | 0.51 |
| APH(6)-Id | 21 | 0.68 | 0.51 |
| bacA | 30 | 0.97 | 0.86 |
| baeR | 31 | 1 | 0.92 |
| baeS | 30 | 0.97 | 0.88 |
| catB3 | 5 | 0.16 | 0.06 |
| catI | 4 | 0.13 | 0.10 |
| CMY-2 | 5 | 0.16 | 0.08 |
| CMY-43 | 1 | 0.03 | 0.00 |
| cpxA | 30 | 0.97 | 0.92 |
| CRP | 31 | 1 | 0.94 |
| CTX-M-1 | 8 | 0.26 | 0.18 |
| CTX-M-117 | 1 | 0.03 | 0.02 |
| CTX-M-14 | 8 | 0.26 | 0.16 |
| CTX-M-15 | 17 | 0.55 | 0.31 |
| CTX-M-27 | 2 | 0.06 | 0.04 |
| CTX-M-28 | 1 | 0.03 | 0.02 |
| CTX-M-3 | 1 | 0.03 | 0.02 |
| CTX-M-55 | 5 | 0.16 | 0.12 |
| CTX-M-60 | 1 | 0.03 | 0.00 |
| CTX-M-79 | 1 | 0.03 | 0.00 |
| dfrA1 | 9 | 0.29 | 0.20 |
| dfrA14 | 5 | 0.16 | 0.08 |
| dfrA17 | 16 | 0.52 | 0.37 |
| dfrA5 | 2 | 0.06 | 0.02 |
| emrA | 30 | 0.97 | 0.90 |
| emrB | 31 | 1 | 0.94 |
| emrK | 29 | 0.94 | 0.86 |
| emrR | 31 | 1 | 0.94 |
| emrY | 29 | 0.94 | 0.86 |
| Enterobacter cloacae acrA | 1 | 0.03 | 0.00 |
| eptA | 30 | 0.97 | 0.90 |
| Escherichia coli acrA | 30 | 0.97 | 0.90 |
| Escherichia coli acrR with mutation conferring multidrug antibiotic resistance | 30 | 0.97 | 0.94 |
| Escherichia coli ampC beta-lactamase | 30 | 0.97 | 0.94 |
| Escherichia coli CyaA with mutation conferring resistance to fosfomycin | 13 | 0.42 | 0.35 |
| Escherichia coli EF-Tu mutants conferring resistance to Pulvomycin | 31 | 1 | 1.00 |
| Escherichia coli emrE | 20 | 0.65 | 0.51 |
| Escherichia coli GlpT with mutation conferring resistance to fosfomycin | 28 | 0.9 | 0.82 |
| Escherichia coli gyrA conferring resistance to fluoroquinolones | 8 | 0.26 | 0.18 |
| Escherichia coli marR mutant conferring antibiotic resistance | 31 | 1 | 0.98 |
| Escherichia coli mdfA | 30 | 0.97 | 0.88 |
| Escherichia coli nfsA mutations conferring resistance to nitrofurantoin | 30 | 0.97 | 0.90 |
| Escherichia coli parC conferring resistance to fluoroquinolone | 15 | 0.48 | 0.31 |
| Escherichia coli PtsI with mutation conferring resistance to fosfomycin | 3 | 0.1 | 0.06 |
| Escherichia coli soxR with mutation conferring antibiotic resistance | 30 | 0.97 | 0.96 |
| Escherichia coli soxS with mutation conferring antibiotic resistance | 30 | 0.97 | 0.96 |
| Escherichia coli UhpT with mutation conferring resistance to fosfomycin | 9 | 0.29 | 0.10 |
| evgA | 29 | 0.94 | 0.86 |
| evgS | 29 | 0.94 | 0.86 |
| floR | 5 | 0.16 | 0.12 |
| FosA2 | 4 | 0.13 | 0.04 |
| FosA3 | 5 | 0.16 | 0.12 |
| FosA4 | 2 | 0.06 | 0.00 |
| gadW | 15 | 0.48 | 0.39 |
| gadX | 29 | 0.94 | 0.92 |
| Haemophilus influenzae PBP3 conferring resistance to beta-lactam antibiotics | 30 | 0.97 | 0.92 |
| H-NS | 31 | 1 | 0.94 |
| kdpE | 30 | 0.97 | 0.86 |
| Klebsiella pneumoniae acrA | 2 | 0.06 | 0.02 |
| Klebsiella pneumoniae OmpK37 | 3 | 0.1 | 0.04 |
| linG | 1 | 0.03 | 0.04 |
| marA | 31 | 1 | 0.90 |
| mdtA | 30 | 0.97 | 0.88 |
| mdtB | 30 | 0.97 | 0.88 |
| mdtC | 30 | 0.97 | 0.88 |
| mdtE | 30 | 0.97 | 0.92 |
| mdtF | 30 | 0.97 | 0.92 |
| mdtG | 30 | 0.97 | 0.88 |
| mdtH | 30 | 0.97 | 0.84 |
| mdtM | 29 | 0.94 | 0.88 |
| mdtN | 30 | 0.97 | 0.88 |
| mdtO | 29 | 0.94 | 0.88 |
| mdtP | 30 | 0.97 | 0.90 |
| mef(B) | 2 | 0.06 | 0.04 |
| Morganella morganii gyrB conferring resistance to fluoroquinolone | 1 | 0.03 | 0.00 |
| mphA | 12 | 0.39 | 0.31 |
| Mrx | 12 | 0.39 | 0.31 |
| msbA | 31 | 1 | 0.94 |
| oqxA | 5 | 0.16 | 0.02 |
| OXA-1 | 6 | 0.19 | 0.08 |
| OXY-4-1 | 1 | 0.03 | 0.00 |
| patA | 31 | 1 | 0.94 |
| PmrF | 30 | 0.97 | 0.90 |
| qacH | 3 | 0.1 | 0.08 |
| QnrB1 | 2 | 0.06 | 0.02 |
| QnrS1 | 6 | 0.19 | 0.14 |
| ramA | 2 | 0.06 | 0.00 |
| Salmonella enterica cmlA | 3 | 0.1 | 0.08 |
| Salmonella enterica gyrA conferring resistance to fluoroquinolones | 15 | 0.48 | 0.29 |
| SAT-2 | 7 | 0.23 | 0.12 |
| Shigella flexneri chloramphenicol acetyltransferase | 3 | 0.1 | 0.04 |
| SHV-178 | 5 | 0.16 | 0.14 |
| SHV-32 | 1 | 0.03 | 0.02 |
| SHV-76 | 1 | 0.03 | 0.00 |
| SHV-83 | 1 | 0.03 | 0.02 |
| sul1 | 13 | 0.42 | 0.33 |
| sul2 | 24 | 0.77 | 0.65 |
| sul3 | 3 | 0.1 | 0.08 |
| TEM-1 | 17 | 0.55 | 0.47 |
| TEM-153 | 2 | 0.06 | 0.04 |
| TEM-156 | 3 | 0.1 | 0.06 |
| tet(B) | 8 | 0.26 | 0.22 |
| tet(C) | 17 | 0.55 | 0.39 |
| tet(D) | 8 | 0.26 | 0.20 |
| tet(J) | 1 | 0.03 | 0.00 |
| tetR | 8 | 0.26 | 0.22 |
| TolC | 30 | 0.97 | 0.92 |
| TRU-1 | 1 | 0.03 | 0.02 |
| ugd | 24 | 0.77 | 0.67 |
| vgaC | 16 | 0.52 | 0.39 |
| YojI | 30 | 0.97 | 0.90 |

**Appendix Figure 1.** Aerial view of the bay of Port-Saint-Louis-du-Rhône and Carteau islet. The large purple star indicates the location of the islet in the bay. The numbered blue dots on the close-up view represent the location of the 30 nests sampled.


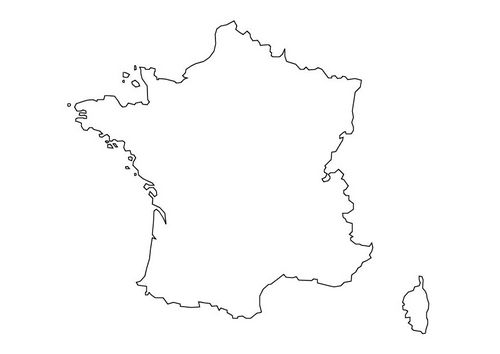


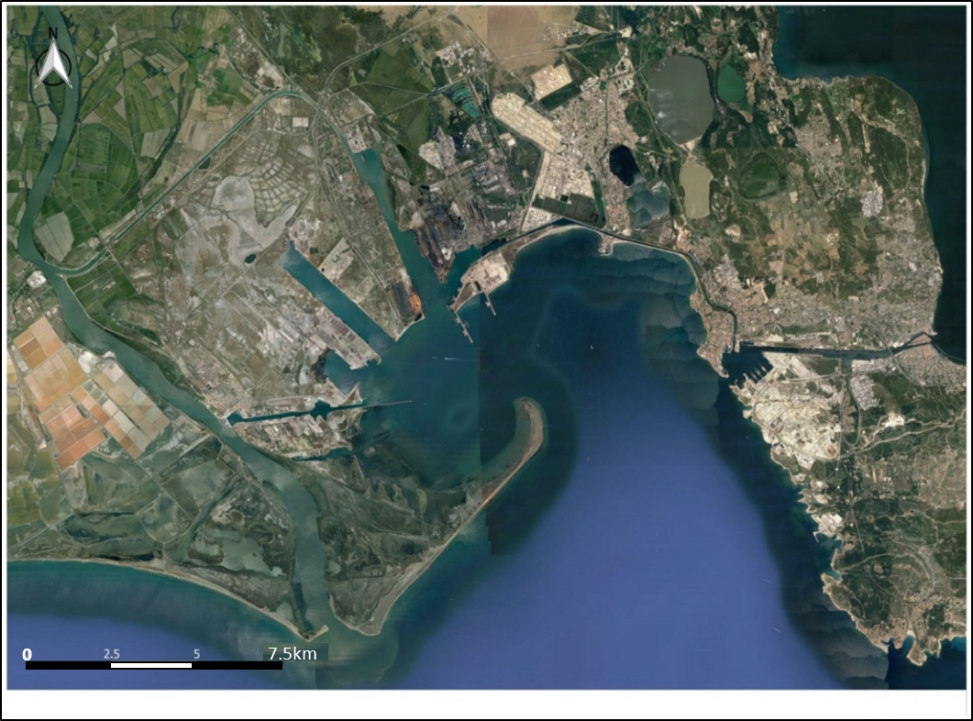


**Port-Saint-Louis-du-Rhône**

**Fos-sur-Mer**

**Port-de-Bouc**

**Martigues**


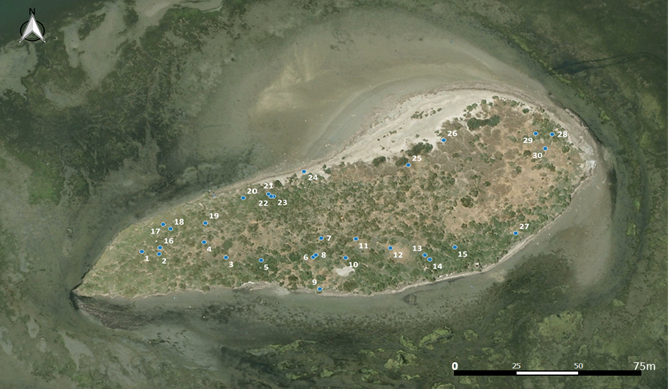


**Carteau islet**

**Appendix Figure 2.** Distribution of numbers of AROs by sampling dates in the ESBL-producing *Enterobacteriaceae* strains pooled.


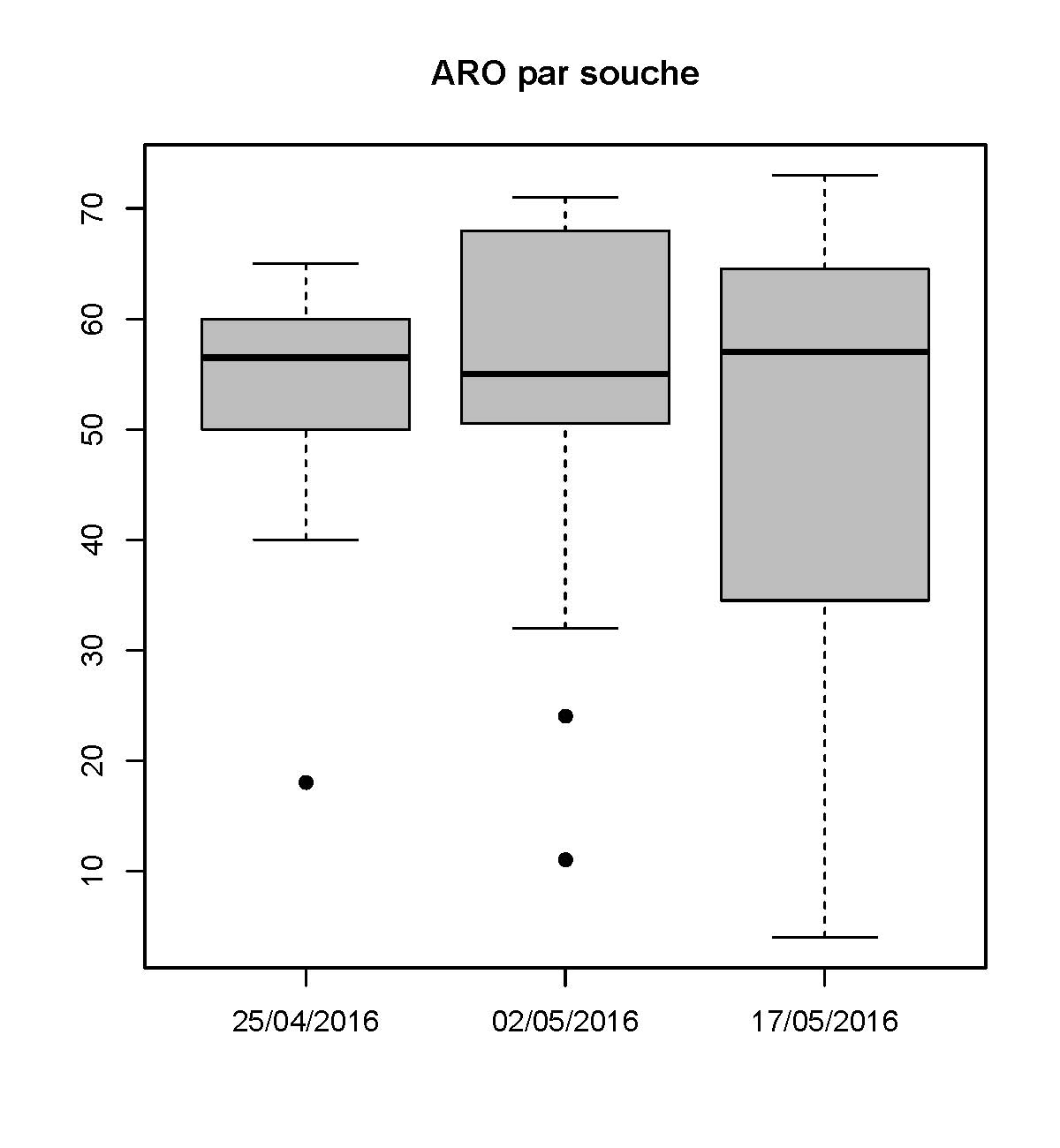


D1

D3

D2

**Appendix Figure 3.** Distributions of the numbers of AROs *E. coli* per species and other species pooled, or per species considered individually.

1. Distributions of the number of AROs in *Escherichia coli* and in other *Enterobacteriaceae*
2. Distribution of the number of AROs per species
3. Distribution of frequency of AROs by sampling date

*Cf: Citrobacter freundii; Ea: Escherichia alberti; Ec: Escherichia coli; Ecl: Enterobacter cloacae; Ef: Escherichia fergusonii; Ek: Enterobacter kobei; Ha: Hafnia alvei; Ka: Klebsiella aerogenes; Kp: Klebsiella pneumoniae; Pm: Proteus mirabilis.*

App. Figure 3-a shows the distribution of the number of AROs in strains of *E. coli* vs strains of non-*E coli* bacteria. *E. coli* bacteria harbour significantly more AROs than non-*E. coli* ones (medians 58 vs 30, Wilcoxon test, P-value=0.0001).

App. Figure 3-b shows the distributions of the number of AROs per species. Among the four *Enterobacteriaceae* species that have many strains, the genomic analysis detected a large number of resistant genes in each strain of *Escherichia coli* (51 strains). In *Klebsiella pneumoniae*, the 4 strains show a great diversity in the number of AROs present. *E. coli* bacteria harbour significantly more AROs than non-*E. coli* ones (median 58 vs 30, Wilcoxon test, P-value=0.0001).

App. Figure 3-c shows the distribution of prevalence of AROs by sampling dates. Irrespective of the sampling date, most genes fall into two categories: those with low (less than 25%) prevalence, and those with high (more than 75%) prevalence, with few genes at intermediate prevalence.


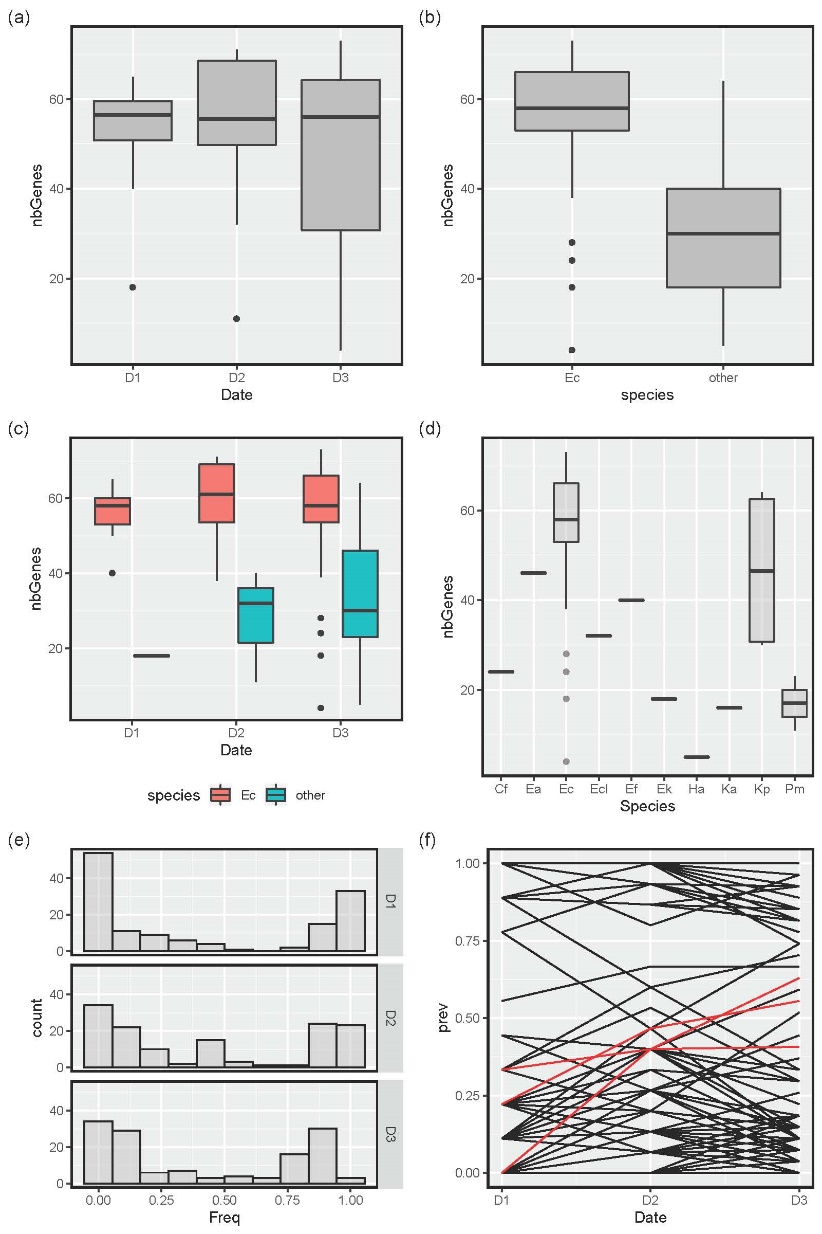

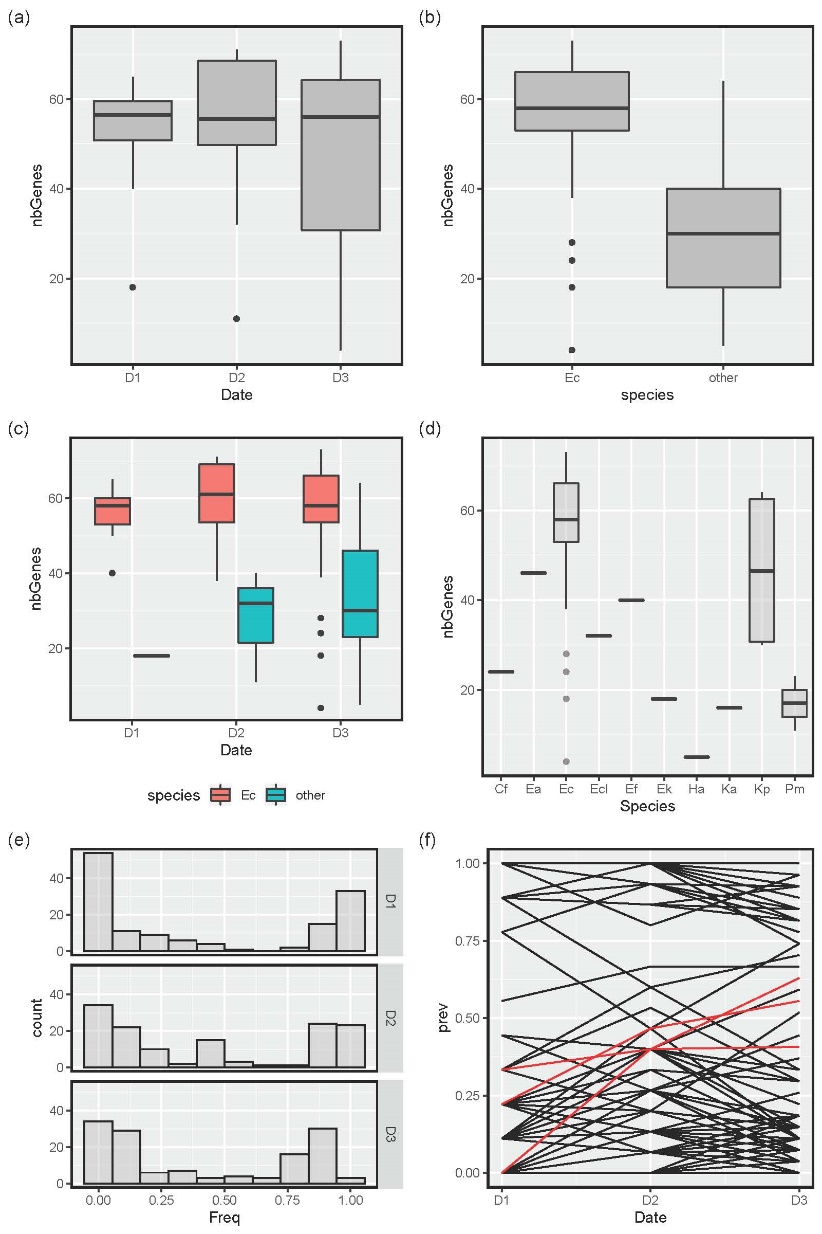

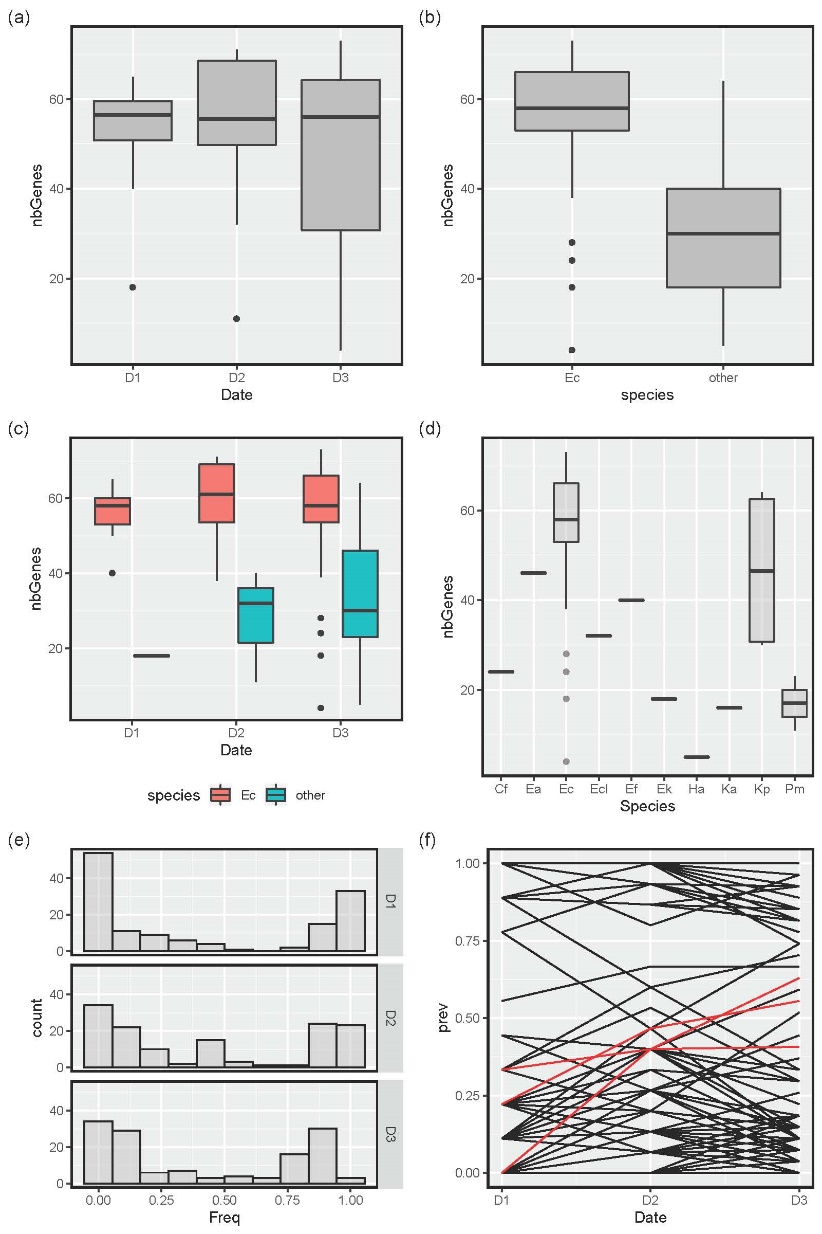


(3-a)

(3-b)

(3-c)

Species

No. ARO

Species

No. ARO

Frequency

Count
